# Adverse Event Reporting with Immune Checkpoint Inhibitors in Older Patients: Age Subgroup Disproportionality Analysis in VigiBase

**DOI:** 10.3390/cancers13051131

**Published:** 2021-03-06

**Authors:** Roberta Noseda, Giulia Bonaldo, Domenico Motola, Anastasios Stathis, Alessandro Ceschi

**Affiliations:** 1 Division of Clinical Pharmacology and Toxicology, Institute of Pharmacological Sciences of Southern Switzerland, Ente Ospedaliero Cantonale, 6900 Lugano, Switzerland; Alessandro.Ceschi@eoc.ch; 2 Unit of Pharmacology, Department of Medical and Surgical Sciences, University of Bologna, 40126 Bologna, Italy; Giulia.Bonaldo2@unibo.it (G.B.); Domenico.Motola@unibo.it (D.M.); 3 Oncology Institute of Southern Switzerland, Ente Ospedaliero Cantonale, 6500 Bellinzona, Switzerland; Anastasios.Stathis@eoc.ch; 4 Faculty of Biomedical Sciences, Università della Svizzera Italiana, 6900 Lugano, Switzerland; 5 Department of Clinical Pharmacology and Toxicology, University Hospital Zurich, 8091 Zurich, Switzerland

**Keywords:** immune checkpoint inhibitors, adverse drug reaction, older patient, VigiBase, disproportionality

## Abstract

**Simple Summary:**

Due to the changes that occur with aging in the immune system, older patients represent a subpopulation of concern for immune checkpoint inhibitor toxicity. This pharmacovigilance study aimed to assess whether older patient age (65 years and older) was a risk factor for increased reporting of adverse drug reactions with immune checkpoint inhibitors as compared to other antineoplastic drugs in VigiBase, the World Health Organization global database of spontaneous reporting. Disproportionality analysis by age subgroups (<18 years, 18–64 years, 65–74 years, 75–84 years and ≥85 years) did not highlight older patient age as risk factor for increased reporting of any specific toxicity with immune checkpoint inhibitors as compared to other antineoplastic drugs. A signal of disproportionate reporting emerged for eye disorders with immune checkpoint inhibitors in patients aged 18–64 years, which deserves further investigation aimed at elucidating risk factors and defining management strategies.

**Abstract:**

Older patients represent a subpopulation of concern for immune checkpoint inhibitor (ICI) toxicity because of changes in the aging immune system and the potentially relevant clinical implications for their quality of life. Current evidence on ICI safety in older patients is conflicting. This study aimed to assess whether older patient age was a risk factor for increased reporting with ICIs as compared to other antineoplastic drugs in VigiBase, the World Health Organization database of suspected adverse drug reactions. Disproportionality analyses computing the reporting odds ratios (RORs) were performed by age subgroups (<18 years, 18–64 years, 65–74 years, 75–84 years and ≥85 years). There were not signals of disproportionate reporting with ICIs specifically detected in older patient age subgroups (≥65 years), which were not present in the disproportionality analysis over the entire dataset. A signal of disproportionate reporting with ICIs emerged for eye disorders only in the age subgroup 18–64 years (ROR 1.13, 95% confidence interval 1.05–1.23). These findings showed that adverse event reporting with ICIs in older patients was comparable to that in the overall patient cohort and prompt for the further investigation of eye disorders with ICIs to elucidating risk factors and defining management strategies.

## 1. Introduction

An increasing number of patients affected by heterogeneous cancer types are currently being treated with immune checkpoint inhibitors (ICIs), due to improved efficacy and better safety profile demonstrated in randomized clinical trials (RCTs) when compared to cytotoxic drugs [1,2]. By enhancing the immune system activity, ICIs are associated with inflammatory side effects, termed immune-related adverse events (irAEs), which may potentially affect any organ [3].

Older patients represent the majority of patients seen in routine cancer practice. Due to the changes that occur with aging in the immune system, with increased levels of autoantibodies and pro-inflammatory cytokines [4], older patients represent a subpopulation of concern for ICI toxicity, which has not been so far exhaustively characterized. Indeed, age itself, comorbidities, use of concomitant drugs, reduced performance status, and impaired functional organ capacity, often constituted criteria for exclusion of older patients from pivotal RCTs that led to ICI approval. Furthermore, even when otherwise eligible, older patients were underrepresented [5,6]. Current evidence from age subgroup analysis in RCTs suggests comparable safety profiles regardless of age [7,8]. Instead, observational studies on ICI toxicity in older patients from daily clinical practice showed contrasting findings, sometimes confirming similar rates of irAEs across age subgroups [9,10,11,12], sometimes suggesting that older patients may be at a higher risk of ICI toxicity [13,14].

In the post-marketing setting, ICI safety profile was comprehensively analysed in the Food and Drug Administration (FDA) Adverse Event Reporting System (FAERS), regardless of patient age, showing signals of disproportionate reporting with ICIs compared to other antineoplastic drugs for endocrine disorders, hepatobiliary disorders, and respiratory, thoracic and mediastinal disorders, amongst others [15]. To date, pharmacovigilance studies with databases of spontaneous reporting focused on older patients treated with ICIs are lacking. Recently, subgroup disproportionality analysis was applied in VigiBase, the World Health Organization (WHO) global database of spontaneously reported suspected adverse drug reactions (ADRs), to characterize signals of disproportionate reporting in groups of patients at higher risk of ADRs, including age subgroups [16].

Due to the expanded use of ICIs and the potentially relevant clinical implications that ICI toxicity could entail for older patient quality of life, further characterization of ICI toxicity in older patients is required. This study aimed to assess whether older patient age was a risk factor for increased reporting with ICIs as compared to other antineoplastic drugs in VigiBase. Therefore, spontaneous reporting of adverse events with ICIs was compared with spontaneous reporting of adverse events with other antineoplastic drugs across age subgroups, with a focus on older age subgroups. Safety reports of ICI-related ADRs were clustered according to patient age (<18 years, 18–64 years, 65–74 years, 75–84 years and ≥85 years), and age subgroup disproportionality analyses were performed using other antineoplastic drugs for comparison. As secondary aim, signals of disproportionate reporting detected in specific age subgroups were described in terms of patient characteristics, ICI treatment, spectrum, time-to-onset and outcome of the ICI-related ADRs of interest.

## 2. Materials and Methods

### 2.1. VigiBase

VigiBase (www.vigiaccess.org, accessed on 30 August 2020) is a global pharmacovigilance database set up by the WHO Collaborating Centre for International Drug Monitoring, Uppsala Monitoring Centre (WHO-UMC) [17]. VigiBase contains over 20 million anonymized safety reports of suspected ADRs submitted by member countries of the WHO Programme for International Drug Monitoring. In the post-marketing setting, health care professionals, patients, pharmaceutical companies, and others, when face with any suspected ADR, can spontaneously report them to national pharmacovigilance centres, which, after revision and analysis, ultimately communicate them to the WHO-UMC to share in VigiBase. VigiBase relies on a medical classification system, the WHO-Medical Dictionary for Regulatory Activities (MedDRA), which arranges information on reported ADRs into a structured and hierarchical form. The “system organ classes” (SOCs) cluster ADRs by manifestation site or aetiology, and “preferred terms” (PTs) describe ADRs as single medical concepts. Information recorded within each safety report comprises administrative features (reporting year, country of origin, type of reporter); patient characteristics (age, sex), reaction (onset and end date, outcome), and drug details (role in ADR onset as attributed by reporter—suspected, interacting, or concomitant -; indication, start and end dates). Lastly, when a safety report is identified by reporters as serious, it is mandatory to select the seriousness criterion as defined by the WHO-UMC (i.e., serious for causing or prolonging hospitalization, leading to death, representing a life-threatening condition, disabling/incapacitating, or determining other clinically relevant conditions) [16].

### 2.2. Study Design

This was a pharmacovigilance study based on age subgroup disproportionality analysis. On 30 August 2020, de-duplicated safety reports gathered in VigiBase since its inception (1968) and concerning ADRs reported with ICIs (i.e., ipilimumab, nivolumab, pembrolizumab, atezolizumab, avelumab, durvalumab, and cemiplimab, all FDA approved, recorded as active ingredients and denoted as suspected) were retrieved. According to patient age, the following subgroups were defined: <18 years, 18–64 years, 65–74 years, 75–84 years, and ≥85 years. Due to the lack of a universally accepted cut-off age to identify older adults [18], older patients were designated as those aged 65 years or older. Demographic information (reporting country and reporting year, reporter qualification, safety report seriousness), patient characteristics (age and sex), and ICI treatment (regimen and indication), were described within each age subgroup. Based on molecular targets, three ICI regimens were distinguished: anti-cytotoxic T-lymphocyte antigen-4 (CTLA-4) monotherapy (i.e., ipilimumab), anti-programmed cell death-1/programmed cell death-ligand 1 (PD-1/PD-L1) monotherapy (i.e., nivolumab, pembrolizumab, cemiplimab as anti-PD-1 drugs; atezolizumab, avelumab, and durvalumab as anti-PD-L1 drugs), and combination of anti-PD-1 with anti-CTLA-4. In the presence of two or more suspected ICIs with partially recorded or missing dates of administration, ICI regimen was not definable.

### 2.3. Disproportionality Analysis

Disproportionality analysis is a statistical method increasingly used in pharmacovigilance studies assessing ICI toxicity in spontaneous reporting systems [19]. In this analysis, the proportion of a certain adverse event/drug combination is compared with the proportion of the same adverse event reported in combination with any other drug recorded in the pharmacovigilance database and with the proportion of other adverse events reported in combination with the drug of interest. For the present study, ICIs were considered against other antineoplastic drugs (of the anatomical, chemical and therapeutic group, ATC, L01). This approach, named disproportionality analysis by therapeutic area, mitigates confounding by indication and provides a clinical perspective allowing for the selection of cancer patients presumably sharing similar risk factors [19]. MedDRA SOCs were used to cluster the ADRs of interest by organs. Due to lack of exposure data, the denominator used in disproportionality analysis is the total number of safety reports for each group of drugs. The reporting odds ratio (ROR) was used as measure of disproportionate reporting and considered statistically significant when at least five safety reports of interest were reported and the lower bound of the 95% confidence interval (CI) for the ROR measurement was >1 [14]. In these conditions, signals of disproportionate reporting indicated that the proportion of a certain adverse event/drug combination was greater in patients exposed to ICIs than in patients not exposed to ICIs. At first, disproportionality analysis was performed on the entire datasets of safety reports associated with either ICIs or other antineoplastic drugs, without age restrictions and including safety reports lacking information on patient age. Then, disproportionality analysis was applied in age subgroups (<18 years, 18–64 years, 65–74 years, 75–84 years, and ≥85 years). Within each age subgroup, RORs were calculated separately and signals of disproportionate reporting counted if the criteria aforementioned were met.

### 2.4. Safety Assessment of Signals of Disproportionate Reporting in Specific Age Subgroups

The secondary aim of the study was the characterisation of signals of disproportionate reporting detected within a specific age subgroup and not in the disproportionality analysis on the entire datasets. Patient characteristics (age, sex, cancer type), spectrum, time-to-onset and outcomes of the ICI-related ADRs of interest, co-reported ICI-related toxicities, and co-suspected drugs, were assessed across ICI regimens. Continuous variables were described as medians and interquartile ranges, categorical variables as counts and percentages. In the description of safety report characteristics, missing data were omitted from the corresponding variables. Differences in the distribution of continuous variables were addressed by the Kruskal–Wallis test. All analysis were carried out by Microsoft Excel (2010, Microsoft Corporation, Washington, DC, USA) and GraphPad Prism (version 9.0.0, GraphPad Software, San Diego, CA, USA, www.graphpad.com, accessed on 30 August 2020). 

According to the Human Research Act 810.30 (status as of 1 January 2020), ethical approval by the local Ethical Committee was not required for this study as it involved anonymised health-related data.

## 3. Results

**Figure 1:**
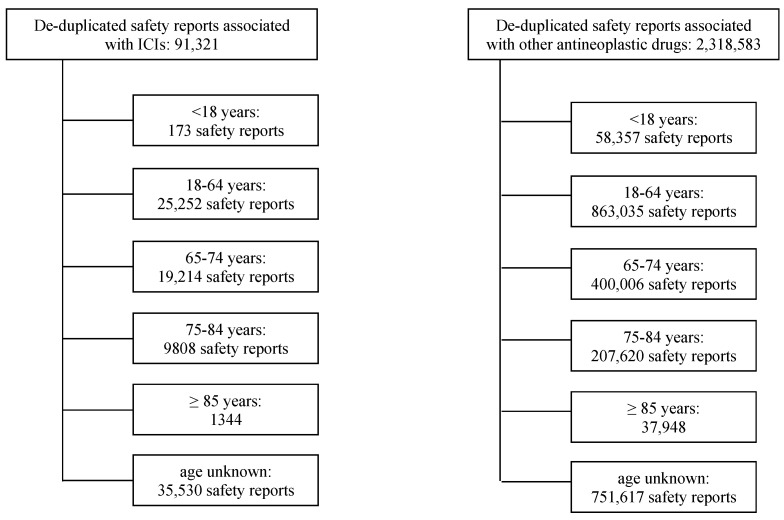
Selection of safety reports associated with either immune checkpoint inhibitors or other antineoplastic drugs, collected in VigiBase from database inception to 30 August 2020. Abbreviations: ICI, immune checkpoint inhibitor.

As of 30 August 2020, 91,321 de-duplicated safety reports of suspected ICI-related ADRs were present in VigiBase (Figure 1). Of these, 35,530 (38.9%) lacked information on patient age, 30,366 (33.3%) involved older patients (designated as those aged 65 years or older), 25,425 (27.8%) involved younger patients (<65 years).

### 3.1. Descriptive Analysis of ICI-Related Safety Reports by Age Subgroups

Table 1 summarizes the baseline characteristics of ICI-related safety reports by age subgroups. In all age subgroups, ICI toxicity was more frequently reported in male patients, ranging from 54.3% in the age subgroup <18 years to 67.6% in the age subgroup 75–84 years. Over the years, reporting of ICI-related ADRs progressively increased in patients aged 18 years or older, with a peak in 2019 ranging from 26.2% in the age subgroup 18–64 years to 31.5% in the age subgroup 75–84 years. Across all age subgroups, physicians were the main reporters (61.8% in the age subgroup 75–84 years), the majority of ICI-related safety reports were serious (78–81%), and the highest number of ICI-related safety reports had an anti-PD-1 monotherapy as suspected for ADR onset (from 61.7% in the age subgroup 18–64 years to 76.7% in the age subgroup ≥85 years). Concerning ICI indication, lung cancer was the main indication in in the age subgroups 18–64, 65–74, and 75–84 years (30.7%, 42.3%, and 40.1%, respectively), malignant melanoma in the age subgroup ≥85 years (31.1%), and malignant melanoma along with haematological malignancy in the youngest age subgroup <18 years (17.9% each).

### 3.2. Disproportionality Analysis

**Figure 2:**
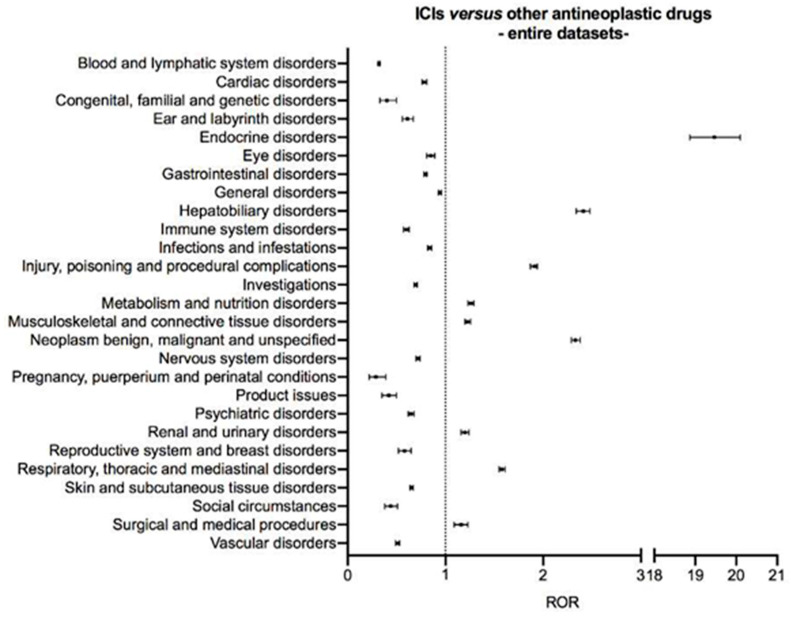
Disproportionality analysis on the entire datasets of safety reports associated with either immune checkpoint inhibitors or other antineoplastic drugs, without age restrictions and including safety reports lacking information on patient age. Suspected adverse drug reactions were clustered according to the Medical Dictionary for Regulatory Activities (MedDRA) System Organ Classes, version 23.0. Vertical bars on the ROR estimates indicate the 95% confidence intervals. Abbreviations: ICI, immune checkpoint inhibitor; ROR, reporting odds ratio.

Disproportionality analysis on the entire datasets of safety reports associated with either ICIs or other antineoplastic drugs, without age restrictions and including safety reports lacking information on patient age, highlighted nine signals of disproportionate reporting: endocrine disorders (ROR 19.47, 95% CI 18.87–20.10), hepatobiliary disorders (ROR 2.41, 95% CI 2.34–2.48), injury, poisoning and procedural complications (ROR 1.91, 95% CI 1.87–1.94), metabolism and nutrition disorders (ROR 1.26, 95% CI 1.23–1.29), musculoskeletal and connective tissue disorders (ROR 1.23, 95% CI 1.20–1.26), neoplasm benign, malignant and unspecified (ROR 2.33, 95% CI 2.29–2.38), renal and urinary disorders (ROR 1.20, 95% CI 1.16–1.24), respiratory, thoracic and mediastinal disorders (ROR 1.58, 95% CI 1.55–1.61), and surgical and medical procedures (ROR 1.16, 95% CI 1.09–1.23) (Figure 2 and Table S1 for details of ROR computation).

**Figure 3:**
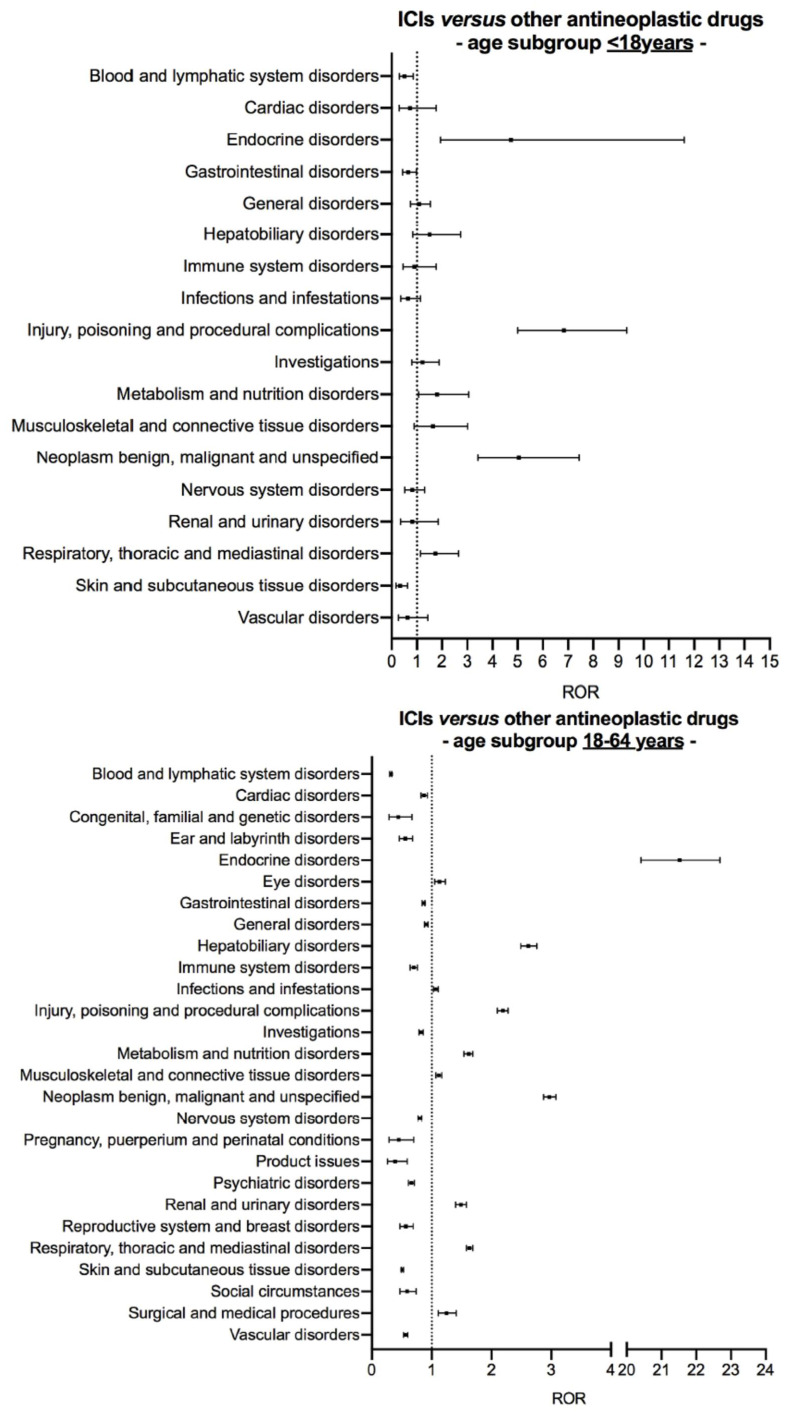
Age subgroup disproportionality analysis of safety reports associated with either immune checkpoint inhibitors or other antineoplastic drugs in younger patients (aged <65 years). Suspected adverse drug reactions were clustered according to the Medical Dictionary for Regulatory Activities (MedDRA) System Organ Classes, version 23.0. Vertical bars on the ROR estimates indicate the 95% confidence intervals. Abbreviations: ICI, immune checkpoint inhibitor; ROR, reporting odds ratio.

Figure 3 shows the results of age subgroup disproportionality analysis comparing reporting of suspected ADRs with ICIs and other antineoplastic drugs in younger patients (<65 years). In the age subgroup <18 years, five of the nine signals of disproportionate reporting previously identified with ICIs on the entire dataset were found: *endocrine disorders* (ROR 4.74, 95% CI 1.94–11.61), injury, poisoning and procedural complications (ROR 6.83, 95% CI 5.00–9.33), metabolism and nutrition disorders (ROR 1.81, 95% CI 1.06–3.07), neoplasm benign, malignant and unspecified (ROR 5.05, 95% CI 3.43–7.43), and respiratory, thoracic and mediastinal disorders (ROR 1.74, 95% CI 1.14–2.65). In the age subgroup 18–64 years, all the nine signals of disproportionate reporting previously identified with ICIs on the entire dataset were confirmed and, in addition, two new signals emerged with ICIs, namely eye disorders (ROR 1.13, 95% CI 1.05–1.23) and infections and infestations (ROR 1.07, 95% CI 1.03–1.11) (see Table S2 for details of ROR computation).

**Figure 4:**
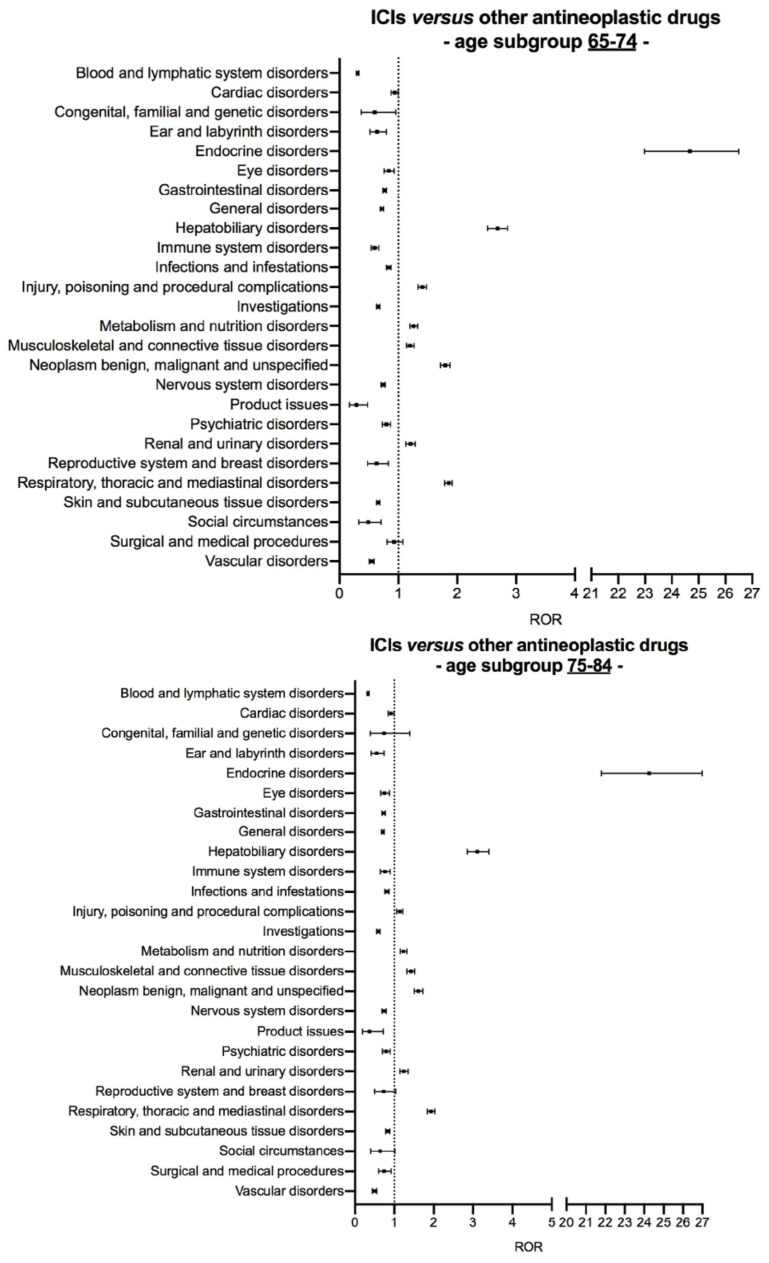

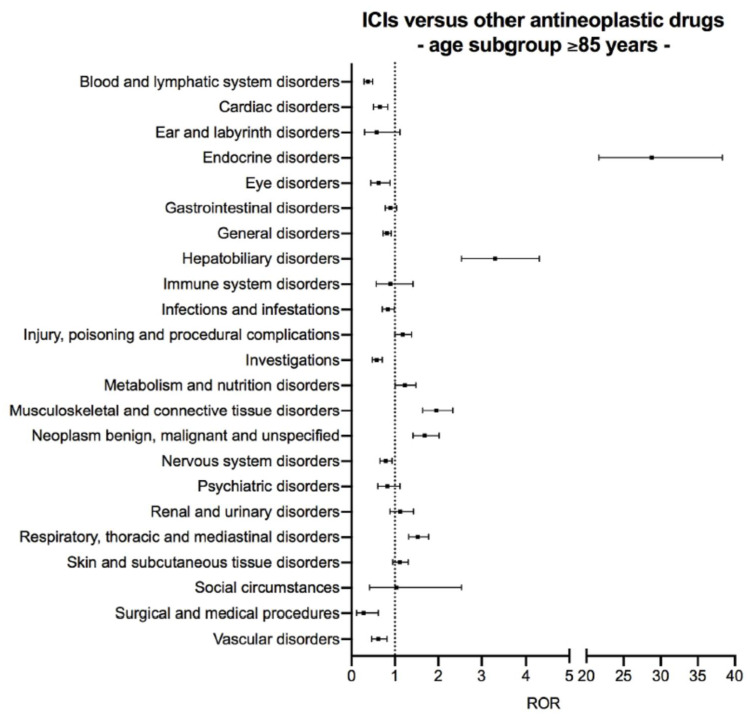
Age subgroup disproportionality analysis of safety reports associated with either immune checkpoint inhibitors or other antineoplastic drugs in older patients (aged ≥65 years). Suspected adverse drug reactions were clustered according to the Medical Dictionary for Regulatory Activities (MedDRA) System Organ Classes, version 23.0. Vertical bars on the ROR estimates indicate the 95% confidence intervals. Abbreviations: ICI, immune checkpoint inhibitor; ROR, reporting odds ratio.

Concerning older patients (≥65 years), signals of disproportionate reporting with ICIs compared to other antineoplastic drugs in the age subgroups 65–74 years and 75–84 years were the same found in the analysis on entire datasets with the exception of *surgical and medical procedures* for which ROR measurements were not statistically significant in both age subgroups (Figure 4). Six out of the nine signals of disproportionate reporting highlighted with ICIs on the entire dataset were found in the age subgroup ≥85 years. These included *endocrine disorders* (ROR 28.82, 95% CI 21.68–38.33), hepatobiliary disorders (ROR 3.31, 95% CI 2.54–4.31), metabolism and nutrition disorders (ROR 1.23, 95% CI 1.01–1.49), musculoskeletal and connective tissue disorders (ROR 1.96, 95% CI 1.64–2.34), neoplasm benign, malignant and unspecified (incl. cysts and polyps) (ROR 1.69, 95% CI 1.42–2.02), and respiratory, thoracic and mediastinal disorders (ROR 1.53, 95% CI 1.32–1.78) (Figure 4 and Table S3 for details of ROR computation).

### 3.3. Signal of Disproportionate Reporting for ICI-Related Eye Disorders in the Age Subgroup 18–64 Years

Eye disorders and infections and infestations were disproportionally more frequently reported with ICIs than with other antineoplastic drugs in the age subgroup 18–64 years (Figure 3 and Table S2). As infections were likely secondary to immunosuppressive therapies used to manage irAEs rather than due to ICI treatment itself, the relative signal of disproportionate reporting was not characterized. Consistently, the use of immunosuppressive therapies may increase the risk of opportunistic infections in patients treated with ICIs [3,20], whilst ICIs per se do not seem to increase such a risk [21]. In the age subgroup 18–64 years (*N* = 25,252), there were 634 (2.5%) safety reports of eye disorders with ICIs, mainly anti-PD-1/PD-L1 agents (*n* = 411, 64.8%, Table 2). Regardless of ICI regimen, 471 (74.3%) safety reports of ICI-related eye disorders co-reported additional toxicities. Co-suspected drugs were reported in 88 (13.9%) safety reports (Table 2). Due to some safety reports with multiple ICI-related eye disorders, the total number of eye disorders was 866. Table 3 shows the spectrum of eye disorders having a relative frequency out of all eye disorders ≥1%. Across ICI regimens, the median time-to-onset of eye disorders did not significantly differ (p = 0.874), and ICI-related eye disorders had mostly recovered (or were recovering) at the time of reporting (327 out of 470 eye disorders for which the outcome was reported, 69.6%, Table 3).

## 4. Discussion

By using a novel subgroup disproportionality approach [16], this study found that patient age was not a risk factor for adverse event reporting with ICIs as compared to other antineoplastic drugs. In VigiBase, there were not signals of disproportionate reporting with ICIs specifically detected in older patients (age ≥65 years) and not present in the disproportionality analysis over the entire dataset (i.e., regardless of patient age and with the inclusion of safety reports missing information on patient age). First, disproportionality analysis over the entire dataset of ICI-related safety reports confirmed previous findings in the FAERS database by Raschi et al. [15], showing a variegate spectrum of ICI-related organ toxicities. Then, age subgroup disproportionality analysis revealed adverse event reporting variability with ICIs across age subgroups. In all older age subgroups, the signal of disproportionate reporting concerning surgical and medical procedures, which was detected in the disproportionality analysis over the entire dataset, did not emerge, likely because the terms included in the correspondent MedDRA SOC refer to adverse events less specifically linked to the immune-related mechanism of action of ICIs. Moreover, the signals of disproportionate reporting for injury, poisoning and procedural complications and renal and urinary disorders seen over the entire dataset, were not detected in patients ≥ 85 years, probably due to the relatively small sample size of the correspondent safety reports with ICIs as compared to those with other antineoplastic drugs in that age subgroup. In patients aged 18–64 years, subgroup disproportionality analysis confirmed the safety profile observed for ICIs in the disproportionality analysis on the entire dataset and highlighted an additional signal of disproportionate reporting for eye disorders, which only emerged in that age subgroup. Ocular adverse events with ICIs are increasingly described in the scientific literature [22,23,24,25]. Recently, two case series of patients who experienced eye toxicity with ICIs pointed out a plethora of ocular manifestations caused by intraocular inflammation, similar to the wide spectrum of eye disorders observed in the present study in patients aged 18–64 years treated with ICIs [22,23]. Consistently with current available information, eye disorders reported in VigiBase with ICIs developed within two months since treatment initiation and had mostly a favourable outcome [24]. Moreover, a previous disproportionality analysis in FAERS showed that reporting with ICIs of uveitis, dry eye, ocular myasthenia and eye inflammation was overall disproportionally higher than with all other drugs [25].

Despite the biological plausibility for suspicious increased risk for ICI toxicity in older patients, due to high levels of autoantibodies and pro-inflammatory cytokines [4], conflicting findings have been reported from either experimental or observational studies [7,8,9,10,11,12,13,14]. A recent review of twelve observational studies in real-life older patients treated with ICIs highlighted that, despite a trend of higher irAEs incidence in older patients, the retrospective nature of the studies analysed and the relatively small numbers of patients included do not allow drawing any definitive conclusion [13]. Dealing with the largest-to-date series of suspected ADRs reported in older patients receiving ICI treatment in clinical practice, the findings from the present study provide further evidence of a comparable safety profile of ICIs regardless of age. 

Disproportionality analysis is a valuable methodological approach well consolidated and extensively used in pharmacovigilance studies on databases of spontaneous reporting to characterize ICI toxicity [19]. Recently, Sandeberg et al. [16], proposed subgroup disproportionality analyses to take into account the diversity that underlines safety reports of different patients, the confounding factors and the potential distortions of disproportionate measurements computed regardless of risk groups for ADRs. Indeed, subgroup disproportionality analysis benefits from both high sensitivity and precision over crude analyses in large databases [26]. Moreover, the choice of using the MedDRA SOC (the highest and more general level), increased the sensitivity of signal detection [27]. 

To properly interpret the results of this study, the limitations of both the data source and the methodological approach must be acknowledged. In VigiBase, safety reports describe suspected ADRs for which, most of the times, the causal relationship with a specific drug cannot be proven, given the presence of comorbidities and concomitant drugs. As regards to comorbidities, these are not systematically collected in VigiBase, which lacks clinical details. Due to this limitation, it was not feasible to perform a sensitivity disproportionality analysis with safety reports of ICI-related eye disorders without pre-existing ocular diseases, which could have helped to assess the role of pre-existing ocular diseases as potential confounding factor of the causal relationship between ICI use and the onset of eye disorders. Since information comes from a variety of sources, the probability that the suspected adverse event is drug-related is not the same in all cases. Moreover, spontaneous reporting is influenced by biases such as selective reporting and underreporting (i.e., lack of safety reports for all the ADRs that actually occur). As the number of safety reports associated with a certain drug (or class of drugs) and reporting a specific ADR can be subject to lower or higher levels of underreporting than the number of safety reports concerning the same ADR with other drugs, the overall number of ADRs occurring with a certain drug (or class of drugs) can be underestimated. As data collection occurs on voluntary basis, it might be partial and incomplete, hence further contributing to distorted estimates of the number of ADRs that occur in clinical practice. As no information is provided on the overall number of patients exposed to a certain drug, the incidence of an ADR of interest cannot be extrapolated. The ROR computation does not inform about the real risk of a certain ADR but only shows an increased risk of ADR reporting. Lack of disproportionality should not to be interpreted as the drug of interest being potentially free of any specific adverse event. As signal detection depends on the number of safety reports, a signal of disproportionate reporting might be detected on larger samples, thus disproportionality analysis should be reassessed at regular intervals for confirmation or rejection.

## 5. Conclusions

Disproportionality analysis by age subgroups in VigiBase did not highlight older patient age as risk factor for increased reporting of any specific toxicity with ICIs as compared to other antineoplastic drugs. This study supports previous findings of comparable safety profile of ICIs regardless of patient age [7,8,9,10,11,12] and adds a paramount piece of knowledge on ICI safety. The largest pharmacovigilance database was used as data source allowing the retrieval and the analysis of the largest-to-date cohort of older patients reporting ADRs in association with ICI treatment. A specific methodological approach that took into account advanced patient age as a potential risk factor for increased reporting with ICIs was applied. Such a methodological strategy, beyond showing that signals of disproportionate reporting with ICIs in older patients overlapped with those detected on the entire dataset of safety reports, regardless of age, shed light on a new signal of disproportionate reporting for eye disorders associated with ICIs in patients aged 18–64 years. Given the increasing number of published case reports and case series and the growing clinicians’ awareness of the spread ICI toxicity, likely also affecting the ocular system [22,23,24,25], the signal herein generated deserves further investigation in prospective RCTs, aiming at elucidating risk factors for the onset of eye disorders and defining management strategies.

## Figures and Tables

**Table 1:** Baseline characteristics of immune checkpoint inhibitor-related safety reports by age subgroups.

Characteristics	<18 Years (*N* = 173)	18–64 Years (*N* = 25,252)	65–74 Years (*N* = 19,214)	75–84 Years (*N* = 9808)	≥85 Years (*N* = 1344)
Median age (IQR), years	12 (5–15)	56 (49–61)	69 (67–72)	78 (76–80)	87 (85–88)
Patient sex					
Male	94 (54.3)	14,530 (57.5)	12,850 (66.9)	6627 (67.6)	828 (61.6)
Female	74 (42.8)	10,236 (40.5)	6038 (31.4)	3009 (30.7)	486 (36.2)
Not reported	5 (2.9)	486 (1.9)	326 (1.7)	172 (1.8)	30 (2.2)
Reporting country					
North America	84 (48.6)	9830 (38.9)	6005 (31.3)	2991 (30.5)	586 (43.6)
South America	16 (9.2)	379 (1.5)	215 (1.1)	97 (1.0)	17 (1.3)
Europe	50 (28.9)	9042 (35.8)	6416 (33.4)	3238 (33.0)	413 (30.7)
Asia	18 (10.4)	5107 (20.2)	5963 (31.0)	3186 (32.5)	278 (20.7)
Oceania	3 (1.7)	734 (2.9)	564 (2.9)	282 (2.9)	49 (3.6)
Africa	2 (1.2)	160 (0.6)	51 (0.3)	14 (0.1)	1 (0.1)
Reporting year					
2008	-	8 (0)	-	-	-
2009	-	26 (0.1)	1 (0)	3 (0)	-
2010	-	32 (0.1)	5 (0)	3 (0)	-
2011	-	114 (0.5)	46 (0.2)	18 (0.2)	1 (0.1)
2012	5 (2.9)	397 (1.6)	185 (1.0)	92 (0.9)	14 (1.0)
2013	1 (0.6)	311 (1.2)	163 (0.8)	59 (0.6)	8 (0.6)
2014	8 (4.6)	696 (2.8)	299 (1.6)	177 (1.8)	29 (2.2)
2015	9 (5.2)	1597 (6.3)	910 (4.7)	397 (4.0)	60 (4.5)
2016	14 (8.1)	2415 (9.6)	1623 (8.4)	766 (7.8)	100 (7.4)
2017	32 (18.5)	4090 (16.2)	3245 (16.9)	1627 (16.6)	220 (16.4)
2018	42 (24.3)	5790 (22.9)	4792 (24.9)	2435 (24.8)	334 (24.9)
2019	34 (19.7)	6613 (26.2)	5739 (29.9)	3092 (31.5)	402 (29.9)
2020 (up to 30 August)	28 (16.2)	3163 (12.5)	2206 (11.5)	1139 (11.6)	176 (13.1)
Reporter qualification					
Physician	71 (41.0)	13,175 (52.2)	11,624 (60.5)	6066 (61.8)	732 (54.5)
Other Health Professional	59 (34.1)	6227 (24.7)	3705 (19.3)	1766 (18.0)	231 (17.2)
Consumer/Non Health Professional	27 (15.6)	3457 (13.7)	1985 (10.3)	958 (9.8)	225 (16.7)
Pharmacist	11 (6.4)	1668 (6.6)	1380 (7.2)	755 (7.7)	112 (8.3)
Lawyer	-	10 (0)	6 (0)	2 (0)	-
Not reported	5 (2.9)	715 (2.8)	514 (2.7)	261 (2.7)	44 (3.3)
Seriousness					
Yes	135 (78.0)	20,291 (80.4)	15,630 (81.3)	7985 (81.4)	1046 (77.8)
Caused/prolonged hospitalization	42 (24.3)	8541 (33.8)	6958 (36.2)	3491 (35.6)	406 (30.2)
Death	29 (16.8)	4214 (16.7)	3226 (16.8)	1838 (18.7)	290 (21.6)
Disabling/incapacitating	2 (1.2)	185 (0.7)	178 (0.9)	84 (0.9)	12 (0.9)
Life threatening	11 (6.4)	1054 (4.2)	873 (4.5)	438 (4.5)	47 (3.5)
Congenital anomaly/birth defect	-	8 (0)	-	1 (0)	-
Other medically important condition	51 (29.5)	6264 (24.8)	4386 (22.8)	2132 (21.7)	290 (21.6)
Unknown	-	25 (0.1)	9 (0)	1 (0)	1 (0.1)
No	34 (19.7)	4445 (17.6)	3203 (16.7)	1642 (16.7)	265 (19.7)
Not reported	4 (2.3)	516 (2.0)	381 (2.0)	181 (1.8)	33 (2.5)
Suspected ICI(s)					
Anti-CTLA-4 monotherapy					
ipilimumab	20 (11.6)	3382 (13.4)	1698 (8.8)	839 (8.6)	119 (8.9)
Anti-PD-1 monotherapy					
nivolumab	71 (41.0)	8960 (35.5)	7247 (37.7)	3548 (36.2)	442 (32.9)
pembrolizumab	49 (28.3)	6591 (26.1)	5748 (29.9)	3379 (34.5)	540 (40.2)
cemiplimab	-	33 (0.1)	47 (0.2)	75 (0.8)	48 (3.6)
Anti-PD-L1 monotherapy					
atezolizumab	8 (4.6)	1993 (7.9)	1674 (8.7)	777 (7.9)	88 (6.5)
avelumab	1 (0.6)	215 (0.9)	214 (1.1)	142 (1.4)	22 (1.6)
durvalumab	2 (1.2)	912 (3.6)	894 (4.7)	380 (3.9)	18 (1.3)
Combination of anti-PD-1 and anti-CTLA-4	13 (7.5)	1826 (7.2)	1039 (5.4)	397 (4.0)	35 (2.6)
≥2 suspected ICIs but regimen not definable ^#^	9 (5.2)	1340 (5.3)	653 (3.4)	271 (2.8)	32 (2.4)
Cancer type					
Lung cancer	11 (6.4)	7740 (30.7)	8127 (42.3)	3933 (40.1)	375 (27.9)
Malignant melanoma	31 (17.9)	6526 (25.8)	3502 (18.2)	2137 (21.8)	418 (31.1)
Renal cell carcinoma	11 (6.4)	1585 (6.3)	1299 (6.8)	570 (5.8)	62 (4.6)
Urothelial carcinoma	2 (1.2)	708 (2.8)	961 (5.0)	685 (7.0)	85 (6.3)
Squamous cell carcinoma of head and neck	-	557 (2.2)	370 (1.9)	154 (1.6)	11 (0.8)
Breast cancer	1 (0.6)	530 (2.1)	108 (0.6)	37 (0.4)	6 (0.4)
Cancer of the female reproductive system	1 (0.6)	454 (1.8)	261 (1.4)	98 (1.0)	6 (0.4)
Haematological malignancy	31 (17.9)	722 (2.9)	335 (1.7)	172 (1.8)	26 (1.9)
Gastrointestinal tract cancer	7 (4.0)	1080 (4.3)	833 (4.3)	356 (3.6)	47 (3.5)
Skin cancer	-	70 (0.3)	50 (0.3)	98 (1.0)	73 (5.4)
Cancer of the male reproductive system	-	104 (0.4)	142 (0.7)	86 (0.9)	15 (1.1)
Brain cancer	18 (10.4)	225 (0.9)	84 (0.4)	18 (0.2)	3 (0.2)
Colon cancer	-	143 (0.6)	49 (0.3)	38 (0.4)	-
Sarcoma	12 (6.9)	132 (0.5)	36 (0.2)	17 (0.2)	1 (0.1)
Thyroid cancer/parathyroid gland cancer	-	74 (0.3)	32 (0.2)	17 (0.2)	5 (0.4)
Mesothelioma	-	64 (0.3)	86 (0.4)	43 (0.4)	6 (0.4)
Neuroendocrine carcinoma	1 (0.6)	47 (0.2)	79 (0.4)	4 (0)	8 (0.6)
Other ῀	1 (0.6)	305 (1.2)	231 (1.2)	139 (1.4)	13 (1.0)
Not reported	46 (26.6)	4186 (16.6)	2629 (13.7)	1206 (12.3)	184 (13.7)

Data are *n* (%). Abbreviations: ICI, immune checkpoint inhibitor; IQR, interquartile range; CTLA-4, cytotoxic T-lymphocyte antigen-4; PD-1, programmed cell death-1; PD-L1, programmed cell death-ligand1. ^#^ Due to ≥2 suspected ICIs with partially recorded or missing dates of administration. ῀ In the five age subgroups. - <18 years: adrenal gland cancer, *n* = 1. - 18–64 years: adrenal gland cancer, thymoma, *n* = 34; nasopharyngeal cancer, *n* = 31; rectal cancer, *n* = 22; Merkel cell carcinoma, *n* = 19; bone cancer, *n* = 18; oral cancer, *n* = 109; salivary gland cancer, *n* = 15; peritoneal neoplasm, *n* = 10; malignant neoplasm of head, face, and neck, *n* = 8; cardiac neoplasm malignant, *n* = 3; lipoma, tracheal cancer, *n* = 1. - 65–74 years: Merkel cell carcinoma, *n* = 47; oral cancer, *n* = 70; rectal cancer, *n* = 20; laryngeal cancer, *n* = 19; bone cancer, *n* = 13; peritoneal neoplasm, *n* = 12; nasopharyngeal cancer, *n* = 11; tracheal cancer, *n* = 10; thymoma, *n* = 8; malignant neoplasm of head, face, and neck, salivary gland cancer, *n* = 7; adrenal gland cancer, *n* = 4; cardiac neoplasm malignant, *n* = 2; ear neoplasm, *n* = 1. - 75–84 years: Merkel cell carcinoma, *n* = 71; oral cancer, *n* = 34; rectal cancer, *n* = 16; bone cancer, salivary gland cancer, *n* = 5; peritoneal neoplasm, *n* = 4; adrenal gland cancer, *n* = 3; thymoma, *n* = 1. - ≥ 85 years: oral cancer, *n* = 7; Merkel cell carcinoma, *n* = 3; laryngeal cancer, *n* = 1; nasopharyngeal cancer, thymoma, *n* = 1.

**Table 2:** Characteristics of safety reports of *eye disorders* reported with immune checkpoint inhibitors in the age subgroup 18–64 years.

ICI-Related Safety Reports of Eye Disorders	Anti-CTLA-4 Monotherapy (*N* = 121)	Anti-PD-1/PD-L1 Monotherapy (*N* = 411)	Combination of Anti-PD-1 and Anti-CTLA-4 (*N* = 65)	≥2 Suspected ICIs but Regimen Not Definable * (*N* = 37)
Median age (IQR), years	52 (46–58)	55 (48–60)	54 (47–59)	56 (52–60)
Patient sex				
Male	65 (53.7)	210 (51.1)	35 (53.8)	21 (56.8)
Female	55 (45.5)	196 (47.7)	30 (46.2)	16 (43.2)
Unknown	1 (0.8)	5 (1.2)	-	-
Cancer type				
Lung cancer	-	127 (30.9)	2 (3.1)	1 (2.7)
Malignant melanoma	94 (77.7)	120 (29.2)	42 (64.6)	27 (73.0)
Renal cell carcinoma	-	33 (8.0)	7 (10.8)	1 (2.7)
Urothelial carcinoma	-	9 (2.2)	-	-
Squamous cell carcinoma of head and neck	-	9 (2.2)	-	-
Breast cancer	-	9 (2.2)	-	-
Cancer of the female reproductive system	-	11 (2.7)	3 (4.6)	1 (2.7)
Haematological malignancy	3 (2.5)	8 (1.9)	1 (1.5)	-
Gastrointestinal tract cancer	-	6 (1.5)	2 (3.1)	-
Cancer of the male reproductive system	-	2 (0.5)	-	-
Brain cancer	1 (0.8)	6 (1.5)	1 (1.5)	-
Colon cancer	-	4 (1.0)	-	-
Sarcoma	-	2 (0.5)	-	1 (2.7)
Thyroid cancer/parathyroid gland cancer	-	1 (0.2)	-	-
Mesothelioma	-	1 (0.2)	-	-
Neuroendocrine carcinoma	-	2 (0.5)	-	-
Nasopharyngeal cancer	-	5 (1.2)	-	-
Salivary gland cancer	-	2 (0.5)	-	-
Thymoma	-	1 (0.2)	-	-
Not reported	23 (19.0)	53 (13.0)	7 (10.8)	6 (16.2)
Safety reports with co-reported ICI-related toxicities	98 (81.0)	293 (71.3)	52 (81.3)	28 (75.7)
Safety reports with co-suspected drugs	15 (12.4)	62 (15.1)	10 (15.4)	1 (2.7)
of which antineoplastic drugs (ATC L01)	4 (3.3)	44 (10.7)	6 (9.2)	1 (2.7)

Data are *n* (%). * Due to ≥2 suspected ICIs with partially recorded or missing dates of administration. Abbreviations: ICI, immune checkpoint inhibitor; IQR, interquartile range; CTLA-4, cytotoxic T-lymphocyte antigen-4; PD-1, programmed cell death-1; PD-L1, programmed cell death-ligand1; ATC, anatomical, therapeutic, chemical.

**Table 3:** Spectrum, time-to-onset, and outcome of eye disorders reported with immune checkpoint inhibitors in the age subgroup 18–64 years.

Eye Disorders ^#^	with Anti-CTLA-4 Monotherapy (*N* = 185)	with Anti-PD-1/PD-L1 Monotherapy (*N* = 549)	with Combination of Anti-PD-1 and Anti-CTLA-4 (*N* = 82)	with ≥2 Suspected ICIs but Regimen Not Definable * (*N* = 50)
Spectrum ^+^				
Uveitis/autoimmune uveitis	19	78	9	5
Visual impairment	16	48	8	1
Vision blurred	14	45	10	2
Dry eye	1	25	6	2
Diplopia	12	16	4	1
Iridocyclitis	6	14	2	8
Ocular hyperaemia	11	12	3	-
Blindness	5	16	2	1
Visual acuity reduced	7	16	1	-
Eyelid ptosis	2	19	2	-
Eye pain	4	9	9	-
Vogt-Koyanagi-Harada disease	3	15	1	2
Papilloedema	3	7	1	5
Lacrimation increased	4	10	1	-
Photophobia	5	7	1	1
Retinal detachment	2	7	-	2
Eye irritation	2	6	1	1
Eye swelling	2	6	1	1
Cataract	2	5	2	-
Time to onset	29 (15.7)	104 (18.9)	12 (14.6)	
Median (IQR), weeks	7.6 (2.6–12.9)	5.8 (2.1–14.0)	5.3 (3.1–8.4)	-
Outcome				
Recovered/recovering	77 (41.6)	202 (36.8)	30 (36.6)	18 (36.0)
Recovered with sequelae	3 (1.6)	11 (2.0)	1 (1.2)	1 (2.0)
Not recovered	13 (7.0)	98 (17.9)	11 (13.4)	5 (10.0)
Fatal	-	3 (0.5)	-	-
Not reported	92 (49.7)	235 (42.8)	40 (48.8)	26 (52.0)

Data are *n* (%). ^#^ Some safety reports reported multiple eye disorders. * Due to ≥2 suspected ICIs with partially recorded or missing dates of administration. ^+^ Eye disorders with a relative frequency out of all eye disorders ≥1%. Abbreviations: ICI, immune checkpoint inhibitor; IQR, interquartile range; CTLA-4, cytotoxic T-lymphocyte antigen-4; PD-1, programmed cell death-1; PD-L1, programmed cell death-ligand1.

## Data Availability

The data presented in this study are openly available from www.vigiaccess.org by applying the inclusion criteria described in the Methods section of the manuscript.

## Supplementary Materials

The following are available online at https://www.mdpi.com/2072-6694/13/5/1131/s1, Table S1: Disproportionality analysis on entire datasets (without age restrictions and including safety reports lacking information on patient age), Table S2: Subgroup disproportionality analysis in younger adults (as of 30 August 2020), Table S3: Disproportionality analysis in older adults by age subgroups (as of 30 August 2020).

## References

[1] Ribas A., Wolchok J.D. (2018). Cancer Immunotherapy Using Checkpoint Blockade. Science.

[2] Robert C., Long G.V., Brady B., Dutriaux C., Maio M., Mortier L., Hassel J.C., Rutkowski P., McNeil C., Kalinka-Warzocha E. (2015). Nivolumab in Previously Untreated Melanoma without BRAF Mutation. N. Engl. J. Med..

[3] Ramos-Casals M., Brahmer J.R., Callahan M.K., Flores-Chávez A., Keegan N., Khamashta M.A., Lambotte O., Mariette X., Prat A., Suárez-Almazor M.E. (2020). Immune-related Adverse Events of Checkpoint Inhibitors. Nat. Rev. Dis. Primers..

[4] Fulop T., Larbi A., Dupuis G., Le Page A., Frost E.H., Cohen A.A., Witkowski J.M., Franceschi C. (2018). Immunosenescence and Inflamm-aging as Two Sides of the Same Coin: Friends or Foes?. Front. Immunol..

[5] O’Connor J.M., Fessele K.L., Steiner J., Seidl-Rathkopf K., Carson K.R., Nussbaum N.C., Yin E.S., Adelson K.B., Presley C.J., Chiang A.C. (2018). Speed of Adoption of Immune Checkpoint Inhibitors of Programmed Cell Death 1 Protein and Comparison of Patient Ages in Clinical Practice Versus Pivotal Clinical Trials. JAMA Oncol..

[6] Dunn C., Wilson A., Sitas F. (2017). Older Cancer Patients in Cancer Clinical Trials are Underrepresented. Systematic Literature Review of Almost 5000 Meta- and Pooled Analyses of Phase III Randomized Trials of Survival from Breast, Prostate and Lung Cancer. Cancer Epidemiol..

[7] Rai R., McQuade J.L., Wang D.Y., Park J.J., Nahar K., Sosman J.A., Beckermann K.E., Haydu L.E., Lo S., Rubinstein S. (2016). 1113PD - Safety and Efficacy of Anti-PD-1 Antibodies in Elderly Patients with Metastatic Melanoma. Ann. Oncol..

[8] Spigel D.R., McCleod M., Jotte R.M., Einhorn L., Horn L., Waterhouse D.M., Creelan B., Babu S., Leighl N.B., Chandler J.C. (2019). Safety, Efficacy, and Patient-reported Health-related Quality of Life and Symptom Burden with Nivolumab in Patients with Advanced Non-small Cell Lung Cancer, Including Patients Aged 70 Years or Older or with Poor Performance Status (CheckMate 153). J. Thorac. Oncol..

[9] Betof A.S., Nipp R.D., Giobbie-Hurder A., Johnpulle R.A.N., Rubin K., Rubinstein S.M., Flaherty K.T., Lawrence D.P., Johnson D.B., Sullivan R.J. (2017). Impact of Age on Outcomes with Immunotherapy for Patients with Melanoma. Oncologist.

[10] Archibald W.J., Victor A.I., Strawderman M.S., Maggiore R.J. (2020). Immune Checkpoint Inhibitors in Older Adults with Melanoma or Cutaneous Malignancies: The Wilmot Cancer Institute Experience. J. Geriatr. Oncol..

[11] Samani A., Zhang S., Spiers L., Mohamed A.A., Merrick S., Tippu Z., Payne M., Faust G., Papa S., Fields P. (2020). Impact of Age on the Toxicity of Immune Checkpoint Inhibition. J. Immunother. Cancer..

[12] Ridolfi L., De Rosa F., Petracci E., Tanda E.T., Marra E., Pigozzo J., Marconcini R., Guida M., Cappellini G.C.A., Gallizzi G. (2020). Anti-PD1 Antibodies in Patients Aged ≥ 75 Years with Metastatic Melanoma: A Retrospective Multicentre Study. J. Geriatr. Oncol..

[13] van Holstein Y., Kapiteijn E., Bastiaannet E., van den Bos F., Portielje J., de Glas N.A. (2019). Efficacy and Adverse Events of Immunotherapy with Checkpoint Inhibitors in Older Patients with Cancer. Drugs Aging..

[14] Baldini C., Romano P.M., Voisin A.L., Danlos F.X., Champiat S., Laghouati S., Kfoury M., Vincent H., Postel-Vinay S., Varga A. (2020). Impact of Aging on Immune-related Adverse Events Generated by Anti-programmed Death (Ligand)PD-(L)1 Therapies. Eur. J. Cancer..

[15] Raschi E., Mazzarella A., Antonazzo I.C., Bendinelli N., Forcesi E., Tuccori M., Moretti U., Poluzzi E., De Ponti F. (2019). Toxicities with Immune Checkpoint Inhibitors: Emerging Priorities from Disproportionality Analysis of the FDA Adverse Event Reporting System. Target Oncol..

[16] Sandberg L., Taavola H., Aoki Y., Chandler R., Norén G.N. (2020). Risk Factor Considerations in Statistical Signal Detection: Using Subgroup Disproportionality to Uncover Risk Groups for Adverse Drug Reactions in VigiBase. Drug Saf..

[17] Lindquist M. (2008). VigiBase, the WHO Global ICSR Database System: Basic Facts. Drug Inf. J..

[18] Califano R., Gomes F., Ackermann C.J., Rafee S., Tsakonas G., Ekman S. (2020). Immune Checkpoint Blockade for Non-small Cell Lung Cancer: What is the Role in the Special Populations?. Eur. J. Cancer..

[19] Raschi E., Gatti M., Gelsomino F., Ardizzoni A., Poluzzi E., De Ponti F. (2020). Lessons to be Learnt from Real-world Studies on Immune-related Adverse Events with Checkpoint Inhibitors: A Clinical Perspective from Pharmacovigilance. Target Oncol..

[20] Del Castillo M., Romero F.A., Argüello E., Kyi C., Postow M.A., Redelman-Sidi G. (2016). The Spectrum of Serious Infections Among Patients Receiving Immune Checkpoint Blockade for the Treatment of Melanoma. Clin. Infect. Dis..

[21] Redelman-Sidi G., Michielin O., Cervera C., Ribi C., Aguado J.M., Fernández-Ruiz M., Manuel O. (2018). ESCMID Study Group for Infections in Compromised Hosts (ESGICH) Consensus Document on the Safety of Targeted and Biological Therapies: An Infectious Diseases Perspective (Immune Checkpoint Inhibitors, Cell Adhesion Inhibitors, Sphingosine-1-phosphate Receptor Modulators and Proteasome Inhibitors). Clin. Microbiol. Infect..

[22] Kim Y.J., Lee J.S., Lee J., Lee S.C., Kim T.I., Byeon S.H., Lee C.S. (2020). Factors Associated with Ocular Adverse Event after Immune Checkpoint Inhibitor Treatment. Cancer Immunol. Immunother..

[23] Noble C.W., Gangaputra S.S., Thompson I.A., Yuan A., Apolo A.B., Lee J.M., Papaliodis G.N., Kodati S., Bishop R., Magone M.T. (2020). Ocular Adverse Events Following Use of Immune Checkpoint Inhibitors for Metastatic Malignancies. Ocul. Immunol. Inflamm..

[24] Dow E.R., Yung M., Tsui E. (2020). Immune Checkpoint Inhibitor-associated Uveitis: Review of Treatments and Outcomes. Ocul. Immunol. Inflamm..

[25] Fang T., Maberley D.A., Etminan M. (2019). Ocular Adverse Events with Immune Checkpoint Inhibitors. J. Curr. Ophthalmol..

[26] Seabroke S., Candore G., Juhlin K., Quarcoo N., Wisniewski A., Arani R., Painter J., Tregunno P., Norén G.N., Slattery J. (2016). Performance of Stratified and Subgrouped Disproportionality Analyses in Spontaneous Databases. Drug Saf..

[27] Pearson R.K., Hauben M., Goldsmith D.I., Gould A.L., Madigan D., O’Hara D.J., Reisinger S.J., Hochberg A.M. (2009). Influence of the MedDRA Hierarchy on Pharmacovigilance Data Mining Results. Int. J. Med. Inform..

