# Supplementary material for: Adverse Event Reporting with Immune Checkpoint Inhibitors in Older Patients: Age Subgroup Disproportionality Analysis in VigiBase
Source: Cancers (Basel). 2021 Mar 6;13(5):1131. doi: 10.3390/cancers13051131 (PMC7961480; doi:10.3390/cancers13051131)
Supplement: Supplementary file 1 [file cancers-13-01131-s001.pdf]

Toxicities (MedDRA SOCs)	ICIs *versus* other antineoplastic drugs, <u>entire datasets</u> (as of August 30th, 2020)				
	**(a)**	**(b)**	**(c)**	**(d)**	**ROR (95% CI)**
Blood and lymphatic system disorders	4518	86803	315515	1911747	0.32 (0.31-0.33)
Cardiac disorders	3664	87657	112629	2114633	0.78 (0.76-0.81)
Congenital, familial and genetic disorders	84	91237	5062	2222200	0.40 (0.33-0.50)
Ear and labyrinth disorders	452	90869	18022	2209240	0.61 (0.56-0.67)
Endocrine disorders	7024	84297	9490	2217772	**19.47 (18.87-20.10)**
Eye disorders	2040	89281	58226	2169036	0.85 (0.81-0.89)
Gastrointestinal disorders	16205	75116	475119	1752143	0.80 (0.78-0.81)
General disorders and administration site conditions	26288	65033	667697	1559565	0.94 (0.93-0.96)
Hepatobiliary disorders	5002	86319	52316	2174946	**2.41 (2.34-2.48)**
Immune system disorders	1654	89667	66307	2160955	0.60 (0.57-0.63)
Infections and infestations	8614	82707	245888	1981374	0.84 (0.82-0.86)
Injury, poisoning and procedural complications	12519	78802	171272	2055990	**1.91 (1.87-1.94)**
Investigations	8515	82806	288259	1939003	0.69 (0.68-0.71)
Metabolism and nutrition disorders	6565	84756	128810	2098452	**1.26 (1.23-1.29)**
Musculoskeletal and connective tissue disorders	7439	83882	149917	2077345	**1.23 (1.20-1.26)**
Neoplasms benign, malignant and unspecified (incl. cysts and polyps)	13491	77830	154031	2073231	**2.33 (2.29-2.38)**
Nervous system disorders	8513	82808	277995	1949267	0.72 (0.70-0.74)
Pregnancy, puerperium and perinatal conditions	45	91276	3753	2223509	0.29 (0.22-0.39)
Product issues	118	91203	6848	2220414	0.42 (0.35-0.50)
Psychiatric disorders	2280	89041	84290	2142972	0.65 (0.62-0.68)
Renal and urinary disorders	3899	87422	80022	2147240	**1.20 (1.16-1.24)**
Reproductive system and breast disorders	336	90985	14005	2213257	0.58 (0.52-0.65)
Respiratory, thoracic and mediastinal disorders	14684	76637	240455	1986807	**1.58 (1.55-1.61)**
Skin and subcutaneous tissue disorders	10087	81234	356538	1870724	0.65 (0.64-0.67)
Social circumstances	175	91146	9648	2217614	0.44 (0.38-0.51)
Surgical and medical procedures	1249	90072	26325	2200937	**1.16 (1.09-1.23)**
Vascular disorders	2950	88371	137541	2089721	0.51 (0.49-0.53)

Table S1 Disproportionality analysis on entire datasets (without age restrictions and including safety reports lacking information on patient age)
(a) Number of safety reports concerning the toxicity of interest reported in association with ICIs
(b) Number of safety reports concerning toxicities other than the one of interest reported in association with ICIs
(c) Number of safety reports concerning the toxicity of interest reported in association with other antineoplastic drugs
(d) Number of safety reports concerning toxicities other than the one of interest reported in association with other antineoplastic drugs
In bold are signals of disproportionate reporting
Abbreviations: MedDRA, medical dictionary for regulatory activities; SOC, system organ class; ICI, immune checkpoint inhibitor; ROR, reporting odds ratio; CI, confidence interval

Toxicities (MedDRA SOCs)	ICIs *versus* other antineoplastic drugs < 18 years					ICIs *versus* other antineoplastic drugs 18-64 years				
	**(a)**	**(b)**	**(c)**	**(d)**	**ROR (95% CI)**	**(a)**	**(b)**	**(c)**	**(d)**	**ROR (95% CI)**
Blood and lymphatic system disorders	16	157	9650	48707	0.51 (0.31-0.86)	1519	23733	145245	717790	0.32 (0.30-0.33)
Cardiac disorders	5	168	2298	56059	0.73 (0.30-1.77)	986	24266	38459	824576	0.87 (0.82-0.93)
Congenital, familial and genetic disorders	2	171	440	57917	NC	23	25229	1778	861257	0.44 (0.29-0.67)
Ear and labyrinth disorders	-	173	538	57819	NA	110	25142	6700	856335	0.56 (0.46-0.68)
Endocrine disorders	5	168	364	57993	**4.74 (1.94-11.61)**	2308	22944	4016	859019	**21.52 (20.41-22.68)**
Eye disorders	4	169	1679	56678	NC	634	24618	19179	843856	**1.13 (1.05-1.23)**
Gastrointestinal disorders	29	144	13667	44690	0.66 (0.44-0.98)	5217	20035	199672	663363	0.87 (0.84-0.89)
General disorders and administration site conditions	39	134	12405	45952	1.08 (0.75-1.54)	6427	18825	235719	627316	0.91 (0.88-0.94)
Hepatobiliary disorders	12	161	2737	55620	1.51 (0.84-2.73)	1711	23541	23269	839766	**2.62 (2.49-2.76)**
Immune system disorders	9	164	3341	55016	0.90 (0.46-1.77)	585	24667	28477	834558	0.70 (0.64-0.76)
Infections and infestations	13	160	6513	51844	0.65 (0.37-1.14)	2614	22638	84199	778836	**1.07 (1.03-1.11)**
Injury, poisoning and procedural complications	63	110	4514	53843	**6.83 (5.00-9.33)**	2835	22417	47168	815867	**2.19 (2.10-2.28)**
Investigations	24	149	6784	51573	1.22 (0.80-1.89)	2547	22705	103746	759289	0.82 (0.79-0.86)
Metabolism and nutrition disorders	15	158	2915	55442	**1.81 (1.06-3.07)**	2045	23207	44649	818386	**1.62 (1.54-1.69)**
Musculoskeletal and connective tissue disorders	11	162	2320	56037	1.64 (0.89-3.02)	1962	23290	60420	802615	**1.12 (1.07-1.17)**
Neoplasms benign, malignant and unspecified (incl. cysts and polyps)	32	141	2509	55848	**5.05 (3.43-7.43)**	3795	21457	48435	814600	**2.97 (2.87-3.08)**
Nervous system disorders	21	152	8383	49974	0.82 (0.52-1.30)	2635	22617	110065	752970	0.80 (0.77-0.83)
Pregnancy, puerperium and perinatal conditions	4	169	177	58180	NC	20	25232	1516	861519	0.45 (0.29-0.70)
Product issues	-	173	141	58216	NA	23	25229	2007	861028	0.39 (0.26-0.59)
Psychiatric disorders	3	170	1877	56480	NC	650	24602	33421	829614	0.66 (0.61-0.71)
Renal and urinary disorders	6	167	2451	55906	0.82 (0.36-1.85)	1105	24147	25715	837320	**1.49 (1.40-1.58)**
Reproductive system and breast disorders	1	172	277	58080	NC	107	25145	6443	856592	0.57 (0.47-0.69)
Respiratory, thoracic and mediastinal disorders	25	148	5175	53182	**1.74 (1.14-2.65)**	4184	21068	93508	769527	**1.63 (1.58-1.69)**
Skin and subcutaneous tissue disorders	11	162	9719	48638	0.34 (0.18-0.63)	2698	22554	164279	698756	0.51 (0.49-0.53)
Social circumstances	-	173	72	58285	NA	73	25179	4244	858791	0.59 (0.47-0.74)
Surgical and medical procedures	1	172	484	57873	NC	298	24954	8158	854877	**1.25 (1.11-1.41)**
Vascular disorders	6	167	3139	55218	0.63 (0.28-1.43)	950	24302	55718	807317	0.57 (0.53-0.60)

Table S2. Subgroup disproportionality analysis in younger adults (as of August 30th, 2020). In bold: signals of disproportionate reporting.

(a) Number of safety reports concerning the toxicity of interest reported in association with ICIs.

(b) Number of safety reports concerning toxicities other than the one of interest reported in association with ICIs.

(c) Number of safety reports concerning the toxicity of interest reported in association with other antineoplastic drugs.

(d) Number of safety reports concerning toxicities other than the one of interest reported in association with other antineoplastic drugs.

Abbreviations: MedDRA, medical dictionary for regulatory activities; SOC, system organ class; ICI, immune checkpoint inhibitor; ROR, reporting odds ratio; CI, confidence interval; NC, not calculated (less than five safety reports); NA, not applicable.

Toxicities (MedDRA SOCs)	ICIs *versus* other antineoplastic drugs 65-74 years					ICIs *versus* other antineoplastic drugs 75-84 years					ICIs *versus* other antineoplastic drugs ≥ 85 years				
	**(a)**	**(b)**	**(c)**	**(d)**	**ROR (95% CI)**	**(a)**	**(b)**	**(c)**	**(d)**	**ROR (95% CI)**	**(a)**	**(b)**	**(c)**	**(d)**	**ROR (95% CI)**
Blood and lymphatic system disorders	1122	18092	67200	332806	0.31 (0.29-0.33)	530	9278	30320	177300	0.33 (0.31-0.36)	61	1283	4214	33734	0.38 (0.29-0.49)
Cardiac disorders	1004	18210	22156	377850	0.94 (0.88-1.00)	591	9217	13641	193979	0.91 (0.84-0.99)	68	1276	2853	35095	0.66 (0.51-0.84)
Congenital, familial and genetic disorders	18	19126	625	399381	0.60 (0.37-0.96)	10	9798	286	207334	0.74 (0.39-1.39)	-	1344	29	37919	NA
Ear and labyrinth disorders	88	19126	2841	397165	0.64 (0.52-0.80)	47	9761	1787	205833	0.55 (0.41-0.74)	9	1335	439	37509	0.58 (0.30-1.12)
Endocrine disorders	1653	17561	1520	398486	**24.68 (22.98-26.50)**	724	9084	680	206940	**24.25 (21.80-26.99)**	96	1248	101	37847	**28.82 (21.68-38.33)**
Eye disorders	386	18828	9538	390468	0.84 (0.76-0.93)	213	9595	5932	201688	0.75 (0.66-0.87)	34	1310	1500	36448	0.63 (0.45-0.89)
Gastrointestinal disorders	3512	15702	90073	309933	0.77 (0.74-0.80)	1647	8161	44850	162770	0.73 (0.69-0.77)	239	1105	7342	30606	0.90 (0.78-1.04)
General disorders and administration site conditions	4256	14958	113079	286927	0.72 (0.70-0.75)	2313	7495	62295	144695	0.71 (0.68-0.74)	408	936	13175	24773	0.82 (0.73-0.92)
Hepatobiliary disorders	1135	18079	9138	390868	**2.69 (2.52-2.86)**	593	9215	4209	203411	**3.11 (2.85-3.40)**	64	1280	565	37383	**3.31 (2.54-4.31)**
Immune system disorders	314	18900	10748	389258	0.60 (0.54-0.67)	152	9656	4212	203408	0.76 (0.65-0.89)	19	1325	598	37350	0.90 (0.57-1.42)
Infections and infestations	2096	17118	50850	349156	0.84 (0.80-0.88)	1118	8690	28576	179044	0.81 (0.76-0.86)	161	1183	5286	32662	0.84 (0.71-0.99)
Injury, poisoning and procedural complications	1728	17486	26231	373775	**1.41 (1.34-1.48)**	906	8902	17003	190617	**1.14 (1.06-1.22)**	169	1175	4131	35161	1.18 (1.00-1.39)
Investigations	1815	17399	54533	345473	0.66 (0.63-0.69)	892	8916	29988	177632	0.59 (0.55-0.64)	116	1228	5286	35759	0.58 (0.48-0.71)
Metabolism and nutrition disorders	1656	17558	27811	372195	**1.26 (1.20-1.33)**	900	8908	15768	191852	**1.23 (1.15-1.32)**	119	1225	2787	35347	**1.23 (1.01-1.49)**
Musculoskeletal and connective tissue disorders	1484	17730	25983	374023	**1.20 (1.14-1.27)**	841	8967	12888	194732	**1.42 (1.32-1.52)**	144	1200	2189	33141	**1.96 (1.64-2.34)**
Neoplasms benign, malignant and unspecified (incl. cysts and polyps)	2388	16826	29218	370788	**1.80 (1.72-1.88)**	1157	8651	15925	191695	**1.61 (1.51-1.72)**	149	1195	2601	37945	**1.69 (1.42-2.02)**

Nervous system disorders	1838	17376	49752	350254	0.74 (0.71-0.78)	946	8862	26366	181254	0.73 (0.69-0.79)	138	1206	4807	37849	0.79 (0.66-0.94)
Pregnancy, puerperium and perinatal conditions	1	19213	36	399970	NC	-	9808	14	207606	NA	-	1344	3	37945	NA
Product issues	15	19199	1088	398918	0.29 (0.17-0.48)	9	9799	510	207110	0.37 (0.19-0.72)	1	1343	99	37849	NC
Psychiatric disorders	512	18702	13266	386740	0.80 (0.73-0.87)	274	9534	7289	200331	0.79 (0.70-0.89)	45	1299	1527	36421	0.83 (0.61-1.12)
Renal and urinary disorders	974	18240	16929	383077	**1.21 (1.13-1.29)**	578	9230	9978	197642	**1.24 (1.14-1.35)**	75	1269	1893	36055	1.13 (0.89-1.43)
Reproductive system and breast disorders	53	19161	1746	398260	0.63 (0.48-0.83)	30	9778	874	206746	0.73 (0.50-1.04)	4	1340	149	37799	NC
Respiratory, thoracic and mediastinal disorders	4112	15102	51147	348859	**1.86 (1.79-1.92)**	2142	7666	26239	181381	**1.93 (1.84-2.03)**	210	1134	4099	33849	**1.53 (1.32-1.78)**
Skin and subcutaneous tissue disorders	2009	17205	59931	340075	0.66 (0.63-0.69)	1119	8689	28006	179614	0.83 (0.78-0.88)	178	1166	4553	33395	1.12 (0.95-1.31)
Social circumstances	28	19186	1195	398811	0.49 (0.33-0.71)	18	9790	599	207021	0.64 (0.40-1.02)	5	1339	136	37812	1.04 (0.42-2.54)
Surgical and medical procedures	195	19019	4356	395650	0.93 (0.81-1.08)	89	9719	2534	205086	0.74 (0.60-0.92)	6	1338	601	37347	0.28 (0.12-0.62)
Vascular disorders	755	18459	27874	372132	0.55 (0.51-0.59)	329	9479	13640	193980	0.49 (0.44-0.55)	50	1294	2230	35718	0.62 (0.47-0.82)

Table S3. Disproportionality analysis in older adults by age subgroups (as of August 30th, 2020). In bold: signals of disproportionate reporting.
(a) Number of safety reports concerning the toxicity of interest reported in association with ICIs.
(b) Number of safety reports concerning toxicities other than the one of interest reported in association with ICIs.
(c) Number of safety reports concerning the toxicity of interest reported in association with other antineoplastic drugs.
(d) Number of safety reports concerning toxicities other than the one of interest reported in association with other antineoplastic drugs.
Abbreviations: MedDRA, medical dictionary for regulatory activities; SOC, system organ class; ICI, immune checkpoint inhibitor; ROR, reporting odds ratio; CI, confidence interval; NC, not calculated (less than five safety reports); NA, not applicable.
